# Kynurenines and Neurofilament Light Chain in Multiple Sclerosis

**DOI:** 10.3389/fnins.2021.658202

**Published:** 2021-05-25

**Authors:** Dániel Pukoli, Helga Polyák, Cecilia Rajda, László Vécsei

**Affiliations:** ^1^Department of Neurology, Faculty of Medicine, University of Szeged, Szeged, Hungary; ^2^Department of Neurology, Vaszary Kolos Hospital, Esztergom, Hungary; ^3^MTA-SZTE Neuroscience Research Group, Department of Neurology, Faculty of Medicine, Interdisciplinary Excellence Centre, University of Szeged, Szeged, Hungary

**Keywords:** multiple sclerosis, kynurenine, neurofilaments, quinolinic acid, glutamate excitotoxicity, mitochondrial dysfunction, neuro-axonal damage, oxidative stress

## Abstract

Multiple sclerosis is an autoimmune, demyelinating, and neurodegenerative disease of the central nervous system. In recent years, it has been proven that the kynurenine system plays a significant role in the development of several nervous system disorders, including multiple sclerosis. Kynurenine pathway metabolites have both neurotoxic and neuroprotective effects. Moreover, the enzymes of the kynurenine pathway play an important role in immunomodulation processes, among others, as well as interacting with neuronal energy balance and various redox reactions. Dysregulation of many of the enzymatic steps in kynurenine pathway and upregulated levels of these metabolites locally in the central nervous system, contribute to the progression of multiple sclerosis pathology. This process can initiate a pathogenic cascade, including microglia activation, glutamate excitotoxicity, chronic oxidative stress or accumulated mitochondrial damage in the axons, that finally disrupt the homeostasis of neurons, leads to destabilization of neuronal cell cytoskeleton, contributes to neuro-axonal damage and neurodegeneration. Neurofilaments are good biomarkers of the neuro-axonal damage and their level reliably indicates the severity of multiple sclerosis and the treatment response. There is increasing evidence that connections exist between the molecules generated in the kynurenine metabolic pathway and the change in neurofilament concentrations. Thus the alterations in the kynurenine pathway may be an important biomarker of the course of multiple sclerosis. In our present review, we report the possible relationship and connection between neurofilaments and the kynurenine system in multiple sclerosis based on the available evidences.

## Introduction

Multiple sclerosis (MS) is an immune-mediated, chronic inflammatory and demyelinating disease of the central nervous system (CNS), which affects both gray and white matter (Bo et al., 2006). Clinically MS appears in different courses, such as relapsing-remitting MS (RRMS) and the progressive form, which can be primary progressive MS (PPMS) or secondary progressive MS (SPMS) (Polman et al., 2011). Characteristic pathological changes of the disease are perivenular inflammatory lesions, which lead to the formation of demyelinating plaques. These inflammatory infiltrates comprise a large number of T-lymphocytes, in addition fewer B- and plasma cells and autoantibodies were also found (Lassmann, 2013; Karussis, 2014). As a consequence of the inflammatory infiltration and oligodendrocytes damage severe demyelination occurs. Axons are usually less affected at the onset of MS, however, irreversible axonal damage occurs as the disease progresses (Trapp et al., 1998). As the pathomechanism of MS is heterogeneous, the inflammatory process can initiate a pathogenic cascade, including microglia activation, chronic oxidative stress or accumulated mitochondrial damage in the axons, that finally leads to neurodegeneration. Moreover the altered mitochondrial function due to chronic cell stress and disruption of electrolyte homeostasis at the end causes neuronal death (Rajda et al., 2015).

Many studies have described the relationship between several points in the kynurenine pathway (KP) during tryptophan (TRP) degradation and the pathomechanism of neurodegenerative diseases, such as MS. TRP is an essential amino acid with complex metabolism and more than 95% is degraded via the KP, while the remaining 5% is metabolized by the serotonin pathway. During the KP (which may be activated by different pro-inflammatory cytokines) several neuroactive and neuroprotective metabolites are formed. This pathway is responsible for the production of nicotinamide adenine dinucleotide (NAD^+^) (Vécsei et al., 2013). Many of the kynurenines such as the *N*-methyl-D-aspartate (NMDA) receptor agonist excitotoxin quinolinic acid (QUIN), the free radical generators 3-hydroxykynurenine (3-HK), 3-hydroxyantranilic acid (3-HAA), and the neuroprotective kynurenic acid (KYNA), picolinic acid (PIC) display neuroactive properties (Chen et al., 2010; Sundaram et al., 2014). This process can initiate a pathogenic cascade, including microglia activation, glutamate excitotoxicity, chronic oxidative stress or accumulated mitochondrial damage in the axons, that finally disrupt the homeostasis of the neuron, destabilizes the neural cell cytoskeleton, contributing to neuro-axonal damage and neurodegeneration [see review (Lovelace et al., 2016)].

Neurofilaments (NFs) are released in the cerebrospinal fluid (CSF) and blood after cell damage, thus they can be considered as an indicator of neuro-axonal damage (Yabe et al., 2001; Fitzner et al., 2015). This protein serves as a good prognostic biomarker for different neurodegenerative disorders including MS, as its level is proportionally elevated with extended neuro-axonal damage (Khalil et al., 2018). There is some evidence that the KP metabolite, QUIN, causes the hyperphosphorylation of neurofilament in neurons and astrocytes, thereby destabilizing their cytoskeleton (Pierozan et al., 2010, 2014, 2015). In our previous study we have found, that the levels of QUIN is associated with the levels of the neuronal cytoskeleton protein, neurofilament light chain (NfL) (Rajda et al., 2020). These results suggest, that on one hand, the kynurenines are highly relevant contributors to neurodegeneration, on the other hand the metabolic profiling of the KP and NfL could potentially serve as useful biomarkers in the MS progression in the future.

In this review we describe how the dysregulation of the KP and their neurotoxic metabolites contribute to the pathological processes in MS. We focus on the cells which produce these metabolites and the mechanism of action how they cause the neuro-axonal damage leading to neurodegeneration.

## Kynurenine Pathway

Previous studies have suggested a role of the altered metabolism of the TRP system in MS (Rejdak et al., 2002; Hartai et al., 2005; Vécsei et al., 2013). In human brain the majority of TRP is metabolized via the KP, however, not all cells of the CNS contain the complete enzymatic pathway. Astrocytes lack the kynurenine-3-monooxygenase (KMO), and oligodendrocytes do not express indolamine-2,3-dioxygenase (IDO-1/IDO-2) and tryptophan 2,3-dioxygenase (TDO), hence these cells do not synthesize neurotoxic QUIN. The complete pathway is present in infiltrating macrophages, activated microglia cells and neurons (Guillemin et al., 2000; Lovelace et al., 2016).

**FIGURE 1:**
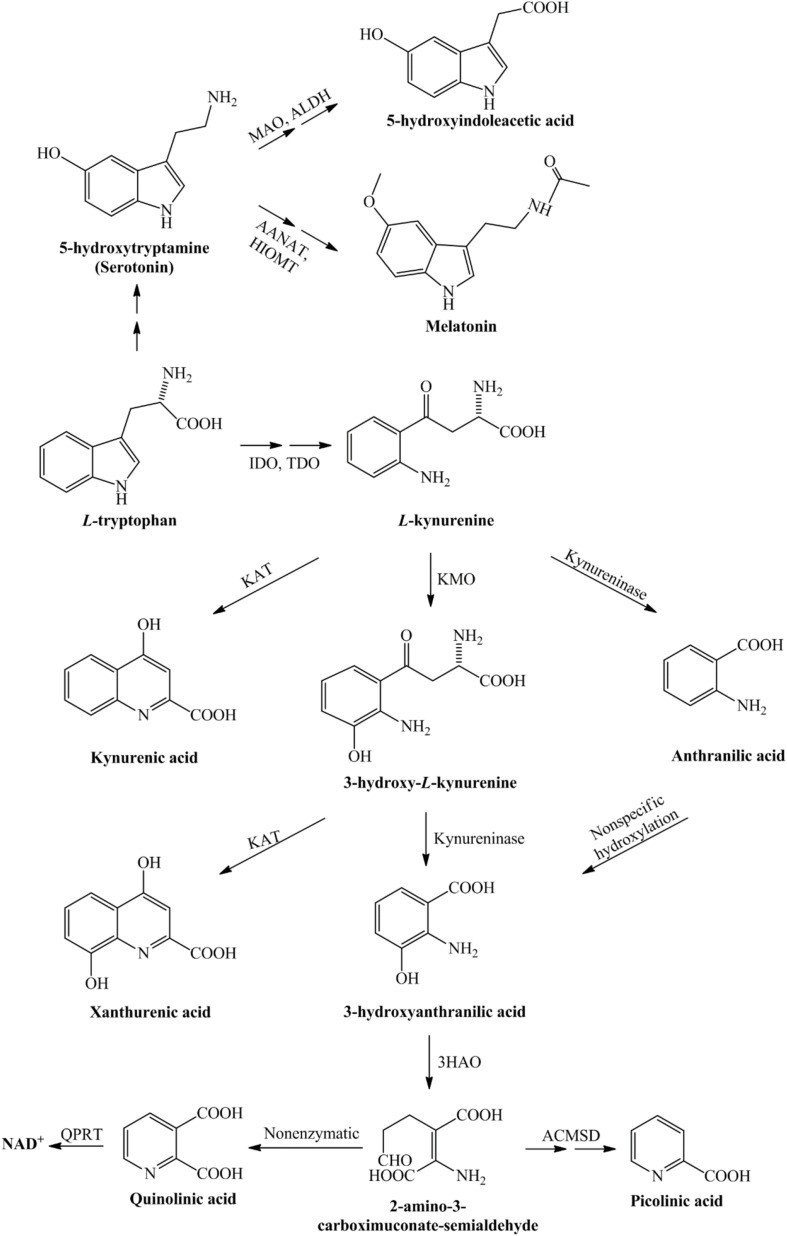
Detailed representation of the kynurenine pathway. Tryptophan, as an essential amino acid, is a precursor of the serotonin- and kynurenine pathway. During the serotonin pathway, melatonin and 5-hydroxyindoleacetic acid are formed, while the kynurenine pathway of tryptophan metabolism leads to the production of nicotinamide adenine dinucleotide. The conversion of tryptophan to L-kynurenine is performed by IDO/TDO. From L-kynurenine various neuroactive metabolites are formed during this pathway, which eventually leads to the nicotinamide adenine dinucleotide, that plays a significant role in the production of cellular energy. 3-HAO, 3-hydroxyanthranilate oxidase; AANAT, arylalkylamine *N*-acetyltransferase; ACMSD, α-amino-β-carboxymuconate-semialdehyde-decarboxylase; ALDH, aldehyde dehydrogenase; HIOMT, hydroxyindole-*O*-methyltransferase; IDO, indoleamine 2,3-dioxygenase; KAT, kynurenine aminotransferase; KMO, kynurenine 3-monooxygenase; MAO, monoamine oxidase; NAD^+^, nicotinamide adenine dinucleotide; QPRT, quinolinate phosphoribosyltransferase; TDO, tryptophan 2,3-dioxygenase.

The first and rate-limiting step in this pathway, the conversion of TRP into L-kynurenine (L-KYN), this process is catalyzed by the TDO, which is mainly localized in liver cells, or the IDO enzyme, which is diffused in most of the human tissues (Sforzini et al., 2019). The KP can be divided into three branches at the L-KYN level, one branch leads to KYNA in an irreversible transamination by kynurenine aminotransferases (KATs), the second route leads to the metabolite that will be metabolized to anthranilic acid or as a third branch it can be degraded to neurotoxic kynurenines in several enzymatic steps. In the human brain, from the four KAT isoforms mainly KAT-II is responsible for KYNA synthesis, which is expressed by astrocytes. In the second branch of the KP, anthranilic acid may be formed from L-KYN by kynureninase. In the third branch of this pathway, L-KYN is transformed to 3-HK by KMO. Further steps in the KP produce 3-HAA from both anthranilic acid by anthranilate 3-monooxygenase and 3-HK by kynureninase. 3-HAA is also capable of autooxidation, it produces cinabarinic acid and generates H_2_O_2_ and superoxide radicals. 3-HAA can be converted via 2-amino-3-carboxymuconate semialdehyde intermediate to PIC by picolinic carboxylase or it can be metabolized by non-enzymatic cyclization to QUIN. Finally, QUIN is metabolized for the synthesis of NAD^+^, via quinolinate phosphoribosyltransferase (QPRT) enzyme [see Figure 1; for review see Bohár et al. (2015)].

## Neuroactive Metabolites

Numerous metabolites of the KP are neuroactive compounds. This section discusses the metabolites with a potential role in the development of MS, including the free radical forming 3-HK, the pro-oxidant, glutamate excitotoxicity inducing, neurotoxic QUIN, and the potentially neuroprotective KYNA.

### 3-HK

3-hydroxykynurenine is formed directly from L-KYN in a reaction catalyzed by KMO enzyme exclusively produced by microglia. Its neurotoxic effect is connected with it’s free radical formation and which increases oxidative stress (Németh et al., 2005). Its concentration is elevated in pathological states [e.g., Parkinson’s disease (PD), Huntington’s disease (HD), and MS] (Vécsei et al., 2013). Its auto-oxidation leads to the formation of hydrogen peroxide and hydroxyl free radicals. However, this process requires the presence of redox active metals (Cu^2+^ and Fe^2+^) and no significant cellular toxicity is expected under physiological conditions (Goldstein et al., 2000). Numerous studies have confirmed that 3-HK has a pro-oxidant effect and generates reactive molecules that induce apoptosis [see review (Colín-González et al., 2013)]. Its oxidation leads to the generation of reactive oxygen species (ROS), resulting in lipid oxidation, protein modification, modulation of inflammatory response and DNA damage and ultimately to cell death. Changes in its concentration can indirectly alter gene expression, DNA repair and intracellular calcium levels (Okuda et al., 1996, 1998). Multiple lines of evidence show that 3-HK is a neurotoxic metabolite and accordingly it may play an important role in the development of neurodegeneration in MS (Vamos et al., 2009). Studies performed in rats with experimental autoimmune encephalomyelitis (EAE) an animal model for MS, demonstrated elevated plasma, brain, and spinal cord 3-HK levels (Chiarugi et al., 2001). Another study evaluated the potential synergistic effect of 3-HK and QUIN in neurotoxicity. In studies of rat brain, the intrastriatal injection of either 3-HK (5 nM) or QUIN (15 nM) caused no or mild damage, while in combination they induced a significant increase in the size of lesions (Colín-González et al., 2013).

### QUIN

Under normal conditions, the brain contains nanomolar concentrations of QUIN (Bohár et al., 2015). In neurodegenerative conditions accompanied by chronic inflammation (e.g., MS) the brain levels of extracellular QUIN increase significantly, primarily due to activated microglia and to a lesser extent due to macrophages penetrating the CNS (Guillemin et al., 2003). Its stimulating effects are exerted via the selective stimulation of the NMDA receptor which can be fully inhibited by NMDA receptor antagonists (Stone and Perkins, 1981). This stimulating effect is not particularly efficient (ED50 > 100 μM) but specific to NR2A and NR2B subunit containing NMDA receptors primarily expressed in the forebrain (striatum and hippocampus) (de Carvalho et al., 1996; Guillemin, 2012). QUIN accumulated in the CNS has a neurotoxic effect acting through multiple mechanisms. In pathological concentration QUIN can activate NMDA receptors resulting in excitotoxicity (Monaghan and Beaton, 1991). Additionally, it can inhibit the re-uptake of glutamate by astrocytes which results in strong neurotoxicity in its microenvironment (Tavares et al., 2002). Thirdly, it can further enhance the toxicity of itself and those of other excitotoxins (e.g., NMDA, glutamate) (Schurr and Rigor, 1993; Guillemin et al., 2005a). Finally, it can decrease glutamine synthetase activity and through this pathway limits the recycling of glutamate to glutamine in astrocytes (Ting et al., 2009). Furthermore, it is known that the complex formation of iron (II) ions with QUIN leads to intense free radical formation (Guillemin et al., 2005b). Its neurotoxic effect contributes to the pathomechanism of MS (Lim et al., 2017).

### KYNA

The neuroprotective kynurenic acid is present in nanomolar concentration in mammalian brain (Moroni et al., 1988). KYNA is an endogenous antagonist of ionotropic glutamate receptors with significant neuroprotective properties (Han et al., 2010). It can affect the glutamatergic transmission in different ways (Zádori et al., 2011): it can be a competitive NMDA receptor antagonist (Kessler et al., 1989) or it behaves as a weak antagonist on the kainate- and α-amino-3-hydroxy-5-methyl-4-isoxazolepropionic acid (AMPA) receptors (Birch et al., 1988). Furthermore, KYNA can inhibit presynaptic α7 nicotinic acetylcholine receptors resulting in regulation of presynaptic glutamate release, which effect contributes greatly to the neuroprotective effect of KYNA (Rajda et al., 2015). Although recent reports suggest this role is failed to repeat to same result (Stone, 2020). KYNA may function as a neuroprotective agent in potential neuroprotective process [for review see Rajda et al. (2015)].

## Evidence of Dysregulation of KP Metabolites in MS

**FIGURE 2:**
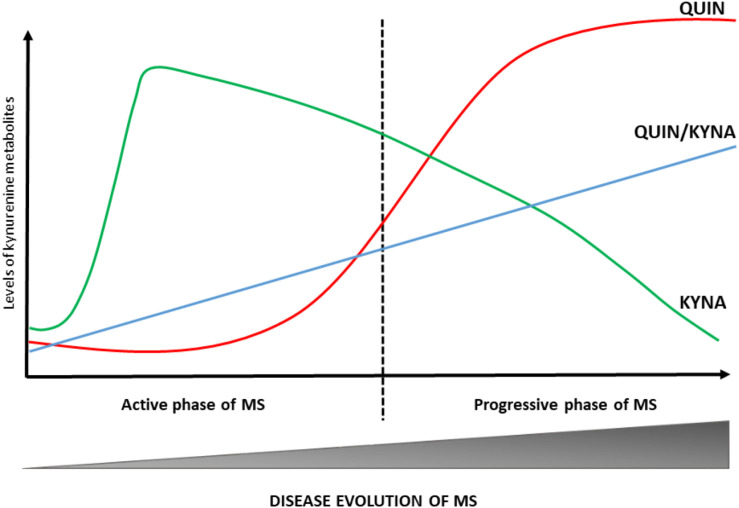
Changes in KP profile during disease course of MS. The KP metabolic profile changes at different stages of MS. In the active phase of MS, the KYNA levels are increased. During the progressive phase of the disease QUIN levels and QUIN/KYNA ratio are increased. These changes in metabolic profile of the KP have a strong association with disease activity. KYNA, kynurenic acid; MS, multiple sclerosis; QUIN, quinolinic acid.

The development and progression of numerous diseases affecting the CNS (including MS) are connected to an enzymatic imbalance of the KP and chronic changes in the physiological concentration of certain kynurenine metabolites (Platten et al., 2005; Lim et al., 2010; Mancuso et al., 2015). The first study, completed 10 years ago, on the potential role of kynurenines in the pathogenesis of MS confirmed lower TRP levels in the blood and CSF of patients with MS (Monaco et al., 1979). Although these results were not supported by a subsequent research, recent studies have demonstrated low blood and CSF TRP concentrations during the chronic phase of MS (Rudzite et al., 1996; Sandyk, 1996). Numerous other studies on changes in KP metabolites have confirmed the activation of KP in MS: Mancuso et al. (2015) verified an increased expression of IDO-1 (the first enzyme of the KP) in patients with RRMS. Furthermore, one of our own study has demonstrated that interferon- β (IFN-β) used to treat MS increases the KYN/TRP ratio, indirectly confirming IDO-1 enzyme mediated activation of the KP in MS (Amirkhani et al., 2005). Rejdak et al. (2002) have confirmed low KYNA level in the CSF of patients with MS. However, two additional studies contradicted these results: Kepplinger et al. (2005) found increased KYNA levels in the CSF of MS patients. In a later study, Rejdak et al. (2007) were found elevated KYNA levels of MS patients in acute relapse. Apart from differences in methodology, these results can be explained by using samples from different phases of disease activity. In particular, the study by Rejdak et al. (2002) was likely carried out in the chronic, inactive phase of the disease, while a more recent study (Kepplinger et al.) analysed samples taken during the patients’ relapse. Hartai et al. (2005) were found elevated KYNA levels in the plasma of MS patients. One study found lower KYNA and PIC levels and increased QUIN levels in MS patients. This neurotoxic level of QUIN is associated with cellular damage in astrocytes, oligodendrocytes and neurons (Vécsei et al., 2013), i.e., disease progression. A comprehensive study examining the connection between KP metabolites and neuro-cognitive symptoms of MS subtypes has detected small differences in KP metabolite levels. RRMS patients demonstrated elevated QUIN levels and higher QUIN/kynurenine ratio during relapse and lower TRP and KYNA levels have been measured in SPMS patients (Aeinehband et al., 2016). These results suggest that the KP is induced during the active phase of MS and leads to increased KYNA production, while during the progressive phase of the disease QUIN levels increase and KYNA levels decrease indicating a change in the KP profile during progression (see Figure 2) (Rejdak et al., 2002, 2007; Hartai et al., 2005). This is further supported by results published by Lim et al., namely that significant aberrations in kynurenine metabolism (divergent levels of two key KP metabolites, KYNA and QUIN) were identified in the blood of MS patients. According to their results, the increased KYNA levels observed in RRMS may constitute a compensatory mechanism working in the initial phase of the disease to counteract neurotoxicity caused by QUIN, and furthermore a moderate correlation can be confirmed between the QUIN/KYNA ratio and the severity of MS. It is the opinion of the authors that changes in kynurenine metabolism may be an important biomarker of the transition from the early, relatively mild form to the progressive form of the disease (Lim et al., 2017).

In summary, alterations of the KP can be found in all phases of the disease (see Table 1). These results suggest that during the early phase of MS (dominated by neuroinflammation) the production of neuroprotective kynurenine metabolites (KYNA and PIC) is the primary mechanism that in all likeliness works to counteract the effects of neurotoxic metabolites. Disease progression brings about a change in KP profile and the chronic activation of the enzymes involved in the pathway enhances the production of neurotoxic metabolites and thus contributes to the emergence of progression in MS (Lim et al., 2010, 2017; Lovelace et al., 2016).

**TABLE 1:** The role of kynurenine metabolites of MS.

**Alterations of the KP in all phases of MS**	**References**
Low KYNA level in the CSF of MS patients in remission	Rejdak et al., 2002
Increased KYNA levels in the CSF of MS patients with acute relapse	Kepplinger et al., 2005; Rejdak et al., 2007
Elevated KYNA levels in the serum of RRMS patients	Lim et al., 2017
Elevated QUIN levels in the CSF of RRMS patients in relapse	Aeinehband et al., 2016
Decreased TRP and KYNA levels in the CSF of SPMS patients	Aeinehband et al., 2016
Elevated QUIN levels in the serum of PPMS patients	Lim et al., 2017

*CSF, cerebrospinal fluid; KP, kynurenine pathway; KYNA, kynurenic acid; MS, multiple sclerosis; PPMS, primary progressive multiple sclerosis; QUIN, quinolinic acid; RRMS, relapsing-remitting multiple sclerosis; SPMS, secondary progressive multiple sclerosis; TRP, tryptophan.*

## Kynurenine Pathway and Immunoregulation of MS

In MS the autoimmune process is mediated by CD4+ Th1 and Th17 cells (Aranami and Yamamura, 2008). At the onset of the disease or in case of a relapse proinflammatory monocytes/macrophages migrate across the damaged blood-brain barrier into the CNS. During the process of neuroinflammation the Th1 cells secrete a variety of proinflammatory cytokines (tumor necrosis factor alpha or TNF-α, interleukin-1, and interleukin-6, etc.) and interferon gamma (IFN-γ) (Sundaram et al., 2020). In the CNS, IFN-γ acts as the primary activating factor for macrophages/microglia cells and enhances their antimicrobial effects by enhancing their production of ROS and nitrogen oxide (NO) and the secretion of various cytokines (interleukin-1 and TNF-α, etc.); and on the other hand, IFN-γ is one of the most potent activators of IDO-1, the primary enzyme of the KP [see review (Braidy and Grant, 2017)]. IDO-1 is present in various immune cells, including monocytes, macrophages, microglia, and may take part in the immunoregulation through TRP depletion and the production of kynurenines (such as KYNA and QUIN) (Mándi and Vécsei, 2012). It’s known, that increased IDO-1 activity suppress the T-cell mediated immune response in MS (Mellor and Munn, 2004; Mancuso et al., 2015), via activation of aryl hydrocarbon receptor (AhR) (Bessede et al., 2014). This has been demonstrated by multiple studies. 3-HAA and QUIN induce the selective apoptosis of Th1 cells (Fallarino et al., 2002). In EAE the pharmacological inhibition of IDO-1 (by the administration of 1-methyl-tryptophan) enhanced the Th1 and Th17 immune response accompanied by decreased regulatory T cells (Treg) response, and the inhibition of IDO-1 exacerbated disease progression. Also, in EAE, 3-HAA suppressed Th1 and Th17 cell activity resulting in enhanced Treg cell formation (Sakurai et al., 2002; Kwidzinski et al., 2005; Yan et al., 2010). As demonstrated, the upregulation of IDO-1 can inhibit the proliferation of autoreactive T cells and promote their selective (Th1 and Th17 cells) apoptosis (Munn et al., 1999; Frumento et al., 2002; Terness et al., 2002). In addition it is capable to induce the formation of immunosuppressive FoxP3 + Treg cells (Fallarino et al., 2006) which inhibit both Th1 and Th2 cells and “assist” in the restoration of a balanced immune system (Mándi and Vécsei, 2012). Several studies explored that the increased kynurenine (by-product of IDO-1) activate the AhR and inhibits the inflammatory response in EAE (Quintana et al., 2010; Mondanelli et al., 2020).

We hypothesize, that in the initial phase of MS–dominated by neuroinflammation–the activation of IDO-1 can be actually beneficial in the short term since it can suppress autoimmune processes via modulation of the T cell mediated immune response (as a negative feedback loop) and promote the development of immune tolerance (Mellor and Munn, 2004; Mancuso et al., 2015). However, in the long term–in the presence of continuous neuroinflammation–chronic IDO-1 activation becomes harmful since the consequential release of chronic neuroactive metabolites contributes to the neurodegeneration observed during MS progression (Lim et al., 2010, 2017).

## Effects of Glial Cells Specificity of KP Metabolites

The primary immune cells of the CNS, microglia cells are highly significant in maintaining the homeostasis of nerve cells. When activated they express multiple pro- and anti-inflammatory cytokines and therefore play an important role in inflammatory and immune response [see review (Luo et al., 2017)]. They also have a significant role in the pathogenesis of MS since during neuroinflammation they damage the myelin sheath and/or oligodendrocytes, activate T cells, and lead to tissue damage, demyelination and cell death, thus contribute to disease progression and neurodegeneration [see review (Luo et al., 2017; Chu et al., 2018)]. During neuroinflammation, pro-inflammatory cytokines induce microglia cells to activate the KP leading to the production of neurotoxic metabolites (QUIN), which play a central role in neuro-axonal damage through various effects such as stimulation of oxidative stress inducers such as ROS, nitric oxide synthase (iNOS) and enhancement of glutamate-mediated neurotoxicity, which can induce neuronal and glial cell death. These effects of the microglia cells can exacerbate neurodegeneration in MS (Lovelace et al., 2017).

Astrocytes are the most abundant cell type in the CNS and play a critical role in the regulation of the blood-brain barrier, providing neuron-glia contact, cellular homeostasis, and glutamate recycling (Guillemin et al., 2001). These cells favor KYNA synthesis as they do not express KMO and therefore they are unable to synthetize neurotoxic metabolites (Guillemin et al., 2001). Rejdak et al. (2007) demonstrated enhanced astrocyte activation in the CSF of MS patients which showed a good correlation with enhanced KYNA production. These results suggest that astrocytes may play a neuroprotective role in MS.

As a result of inflammation in MS, the oligodendrocytes generating the myelin sheath sustain damage which in time leads to axonal degeneration and later to neurodegeneration (Itoh, 2015). Human oligodendrocytes may be important in neuroprotection. During neuroinflammation they provide neuroprotection by generating KYNA and they are capable of taking up exogenous KYN as a substrate for production of downstream KP metabolites causing increased NAD^+^ synthesis. Oligodendrocytes express multiple types of glutamate receptors, such as NMDA, AMPA, and kainate, and are constitutively involved in glutamate clearance (Lim et al., 2010). It is known that during the active phase of MS, the large amounts of neurotoxic QUIN produced by activated macrophages and microglia cells damage oligodendrocytes in the inflammatory microenvironment (Lim et al., 2017), and lead to demyelination followed by neuro-axonal damage.

## Mechanism of Neuro-Axonal Damage of Quinolinic Acid in MS

**FIGURE 3:**
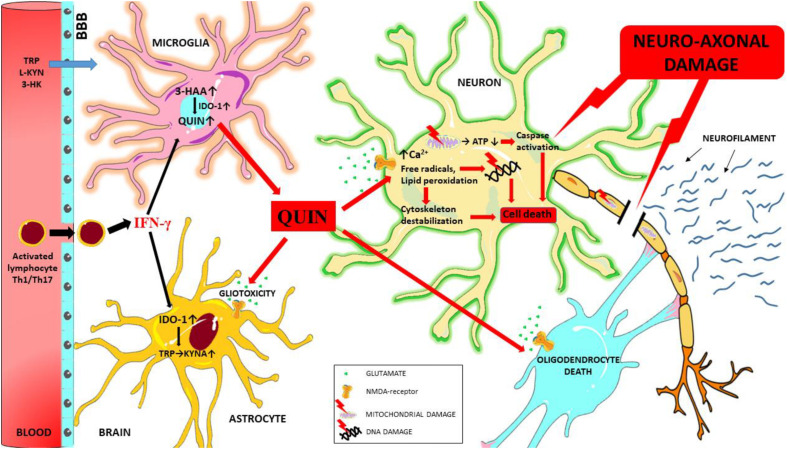
Schematic overview of the mechanisms of QUIN toxicity in MS. During neuroinflammation autoreactive T cells (Th1 and Th17) and activated monocytes/macrophages migrate to the central nervous system through the damaged blood-brain barrier, and these cells secrete numerous pro-inflammatory cytokines (such as interferon gamma). Interferon gamma is one of the most potent activators of IDO-1, the primary enzyme of the KP. In case of an enzymatic imbalance in the KP and also through the chronic alteration of the physiological concentration of kynurenine metabolites in the microenvironment the balance between neuroprotective and neurotoxic processes is no longer ensured. Neurotoxic QUIN secreted in abnormal quantities by activated macrophages and microglia cells in an inflammatory microenvironment can damage oligodendrocytes, astrocytes and neuron through multiple mechanisms. QUIN induces caspase activation, mitochondrial impairment, oxidative stress, lipid peroxidation and energy deficit through the overactivation of NMDA receptors (and also independently of overactivation). These factors combine to result in the destabilization of the cytoskeleton, leading to DNA damage, cell death and ultimately neuro-axonal damage. Neurofilaments are released as a result of neuro-axonal injury. 3-HAA, 3-hydroxyantranilic acid; 3-HK, 3-hydroxykynurenine; ATP, adenosine triphosphate; BBB, blood-brain barrier; IFN-γ, interferon gamma; IDO-1, indoleamine 2,3-dioxygenase; L-KYN, L-kynurenine; KYNA, kynurenic acid; QUIN, quinolinic acid; TRP, tryptophan.

The pro-inflammatory cytokines produced during neuroinflammation can influence IDO-1 activation and thus significantly modify kynurenine metabolism. The neurotoxic metabolites produced during this process (primarily QUIN) can act through multiple pathways to induce neuro-axonal damage (see Figure 3) and ultimately contribute to the process of neurodegeneration observed in MS.

In the next paragraphs, we give insight in some of the most important mechanism of toxicity of QUIN in MS, which process finally lead to neuro-axonal damage.

### Glutamate Excitotoxicity

Excitotoxicity is a pathological process during which the overactivation of excitatory amino acid receptors leads to neuronal damage and death. Excitatory amino acids are the primary excitatory neurotransmitters of the hippocampus and the cerebral cortex and therefore play an essential role in the physiological function of neurons. When it comes to neuronal excitotoxicity it is primarily due to overexposure to glutamate which is the most important excitatory neurotransmitter of the CNS (Mehta et al., 2013).

Glutamate excitotoxicity observed at all stages of MS and might have a substantial role in the neuro-axonal damage, also include progression of MS [see review (Rajda et al., 2017)]. The most important sources of extracellular glutamate are activated microglia/macrophage cells and leukocytes (Stojanovic et al., 2014). Inflammation is triggered by the release of a large amount of glutamate by activated microglia cells [which are activated in all subtypes of MS (Sriram and Rodriguez, 1997)], ultimately leading to nerve cell excitotoxicity and death (Piani et al., 1991; Stojanovic et al., 2014). Glutamate activates NMDA, AMPA, and kainate receptors as well as metabotropic glutamate receptors (Lau and Tymianski, 2010). The NMDA receptor is the central mediator of glutamate excitotoxicity (Fuchs et al., 2012) which is found on the surface of neurons, astrocytes, oligodendrocytes, and microglia cells in the CNS (Kaindl et al., 2012). Its activation or in other words the opening of the channel requires not only glutamate binding but glycine or D-serine binding as well (Papouin et al., 2012).

It is known that QUIN is a selective agonist of NMDA receptors, specifically their NR2A and NR2B subunits (de Carvalho et al., 1996), and can also be considered an endogenous excitotoxin (Stone and Perkins, 1981). Several studies have already proven the role of QUIN in the pathogenesis of MS. Pathologically high levels of QUIN were measured in EAE and in MS patients (Sundaram et al., 2014). Moreover, locally elevated QUIN levels may contribute to demyelination in MS and in EAE (Füvesi et al., 2012). In EAE, exposure to QUIN led to the apoptosis of oligodendrocytes, microglia cells, astrocytes, and neurons (Cammer, 2001). According to the results of Flanagan et al. (1995) a causal relationship can be demonstrated between the degree of clinical severity of the EAE model and QUIN levels measured in the spinal cord. Studies using hippocampus and astrocyte cultures showed that QUIN enhances glutamate release in the synapses, inhibits reuptake and reduces glutamate to glutamine conversion by inhibiting glutamine synthetase. All these effects lead to an increase of synaptic glutamate concentration and eventually to excitotoxicity via the further overstimulation of NMDA receptors (Tavares et al., 2000, 2002; Ting et al., 2009), resulting in an increase of intracellular Ca^2+^ levels. Elevated intracellular Ca^2+^ levels in turn lead to increased neurotransmitter release and the activation of various enzymes. Ca^2+^ ions activate calpains and lead to proteolysis in the cells. Calpain induced proteolysis primarily affects cytoskeletal and signal transduction proteins and transcription factors (Goll et al., 2003). Additionally, phospholipase A2 (PLA2) and cyclooxygenase activation are also enhanced leading to free radical formation followed by lipid peroxidation (Farooqui et al., 1997), and compromised mitochondrial function which in turn causes the formation of ROS and leads to oxidative stress and ultimately to apoptotic cell death (Hardingham, 2009; Sekine et al., 2015). This induced cell death has been observed under *in vitro* conditions in rat oligodendrocytes treated with pathological concentrations of QUIN (1 mM), primary human neurons and astrocytes (at 150 nM QUIN) and motor neurons (at 100 nM QUIN) (Cammer, 2001, 2002; Braidy et al., 2009; Chen et al., 2011).

### Mitochondrial Dysfunction and Oxidative Stress

It has been firmly established that mitochondrial damage and free radicals, such as ROS, and reactive nitrogen species (RNS) play an important role in the pathomechanism and progression of neurodegenerative and inflammatory conditions including MS. Several studies have confirmed that during MS relapse the elevation of free radical concentrations are accompanied by significant changes in the blood concentrations of antioxidant enzymes/reducing agents (Karg et al., 1999; Rajda et al., 2017). Furthermore, free radicals are involved in the maintenance of chronic neuroinflammation, damage the blood-brain barrier and thus promote the migration of immune cells to the CNS, and promote the secretion of pro-inflammatory cytokines. Activated microglia and the pro-inflammatory cytokines, ROS and reactive NO released by macrophages lead to extensive tissue damage, demyelination and cell death, and thus contribute to the mechanism of neurodegeneration in MS (Ohl et al., 2016; Chu et al., 2018).

Through the respiratory chain, mitochondria play important role in cellular energy supply (ATP synthesis), fatty acid metabolism and programmed cell death (apoptosis). Impairment of mitochondrial function causes ATP deficit followed by energy deficit which impairs the function of Na^+^/K^+^-ATPase and Na^+^/Ca^2+^ transporter and consequently leads to membrane depolarization. As a consequence of abnormal membrane potential, cells are more prone to excitotoxic and oxidative damage (Novelli et al., 1988; Sas et al., 2007), and mitochondrial dysfunction results in uncontrolled release of free radicals (ROS and RNS) (Holmström and Finkel, 2014).

We have described above that QUIN can lead to mitochondrial dysfunction via overactivation of NMDA receptors. Recent evidence shows that metabolic impairment in mitochondria is an important mechanism of the manifestation of QUIN toxicity. It has been demonstrated that QUIN can inhibit monoamine oxidase B (MAO-B) in human brain synaptosomal mitochondria (Naoi et al., 1987). QUIN can potentiate its own toxicity and that of other excitotoxins, such as NMDA and glutamate, and thus produce progressive mitochondrial dysfunction (Bordelon et al., 1997). Various studies have shown that the intrastriatal injection of QUIN impairs cellular respiration and induces a reduction of ATP levels (Bordelon et al., 1997, 1998). Ribeiro et al. (2006) have observed that the injection of QUIN in nanomolar concentrations also inhibited creatine kinase activity, an important enzyme involved in intracellular energy transfer. Additionally, QUIN induced a significant reduction of the activity of respiratory chain complexes II (50%), II–III (35%), and III (46%) in the striatum homogenates of juvenile rats. This effect occurred 12 h after QUIN administration. It is possible that these results are primarily due to the activation of glutamate receptors and secondarily to the effect of free radicals induced by QUIN on energy production (Pérez-Severiano et al., 1998; Cabrera et al., 2000; Ganzella et al., 2006). Furthermore, QUIN inhibits around 35% succinate dehydrogenase (SDH), an enzyme involved in the citric acid cycle and in the respiratory chain. Additionally, this effect is independent on the NMDA receptor since MK-801 and KYNA (two NMDA receptor antagonists) and L-NG-nitroarginine methyl ester (L-NAME), a NOS inhibitor, did not prevent the inhibitory effect, but preincubation with superoxide dismutase and catalase did (Schuck et al., 2007). This evidence suggests that the neurotoxic effects of QUIN may be independent of NMDA receptor activity and mitochondrial impairment may be caused by an alternative mechanism of QUIN induced toxicity.

Under physiological conditions (during oxidative phosphorylation) significant amounts of free radicals are produced (ROS and RNS) and they are kept under control by both enzymatic and non-enzymatic antioxidant systems, thus ensuring the redox balance of the body in cells and tissues. The most important enzymatic antioxidants are superoxide dismutase (SOD), glutathione peroxidase (GSH-Px), glutathione reductase (GR), and catalase (Sindhu et al., 2005). This process is in an exquisitely delicate balance and its imbalance leads to oxidative stress and ultimately to cell death. Neurons are especially sensitive to oxidative damage due to their high energy requirements supporting the maintenance of membrane potential, the restoration of ion gradients after action potentials, the release of neurotransmitters and the re-uptake of neurotransmitters from the synaptic gap (Bohár et al., 2015). It is also important to note with respect to neurons that axonal and synaptic mitochondria are more sensitive to oxidative damage than those in dendrites and the cell body (Borrás et al., 2010; Lores-Arnaiz and Bustamante, 2011), suggesting that oxidative damage may also play a role in neuro-axonal damage.

Quinolinic acid induced toxicity includes the generations of free radicals and oxidative stress. In connection with this, it has been shown that QUIN can produce oxidative damage independent of its effects exerted via the NMDA receptor; this mechanism involves the formation of a complex of QUIN and Fe^2+^. The QUIN-Fe^2+^ complex has been shown to induce intensive free radical formation likely responsible for *in vitro* lipid peroxidation and DNA damage (Goda et al., 1996). Additionally, there is evidence that QUIN can enhance free radical production through the induction of NOS activity in astrocytes and neurons which also leads to oxidative stress (Braidy et al., 2010): during neuroinflammation, activated microglia and QUIN produced by macrophages can enhance–via the activation of the NMDA receptor–the activity of NOS enzyme activity in astrocytes and neurons resulting in enhanced nitric oxide (NO) production [see review (Lugo-Huitrón et al., 2013)]. It is important to note that the activated microglia and macrophages themselves can enhance NOS activity (Su et al., 2009). High NO concentration is toxic for cells, since the reaction between NO and oxygen radicals results in the formation of peroxynitrite (ONOO-). NO and ONOO- damage cellular components, inhibit the mitochondrial respiratory chain, cause DNA damage through the overactivation of poly(ADP-ribose) polymerase (PARP) enzymes and enhances extracellular lactate dehydrogenase (LDH) activity and oxidative stress (Sas et al., 2007; Pérez-De La Cruz et al., 2010; Lugo-Huitrón et al., 2013). Overactivation of the PARP enzymes causes free radical accumulation which in turn ultimately leads to cell death through NAD^+^ depletion [see review (Braidy and Grant, 2017)].

Furthermore, it has been shown that in rat brain QUIN is able to modify the profile of certain endogenous antioxidants, for example, it lowers reduced glutathione content and copper and zinc dependent SOD activity (Cu^2+^ and Zn-SOD) (Rodríguez-Martínez et al., 2000). In rat brain the intracerebral injection of QUIN induced a significant decrease in nerve cell numbers and a marked increase in SOD1 expression (Noack et al., 1998). The increase in SOD1 expression was likely a neuroprotective effect limiting oxidative damage caused by QUIN. Based on the above evidence and taking into account that oxidative stress is caused by an imbalance between antioxidant protection and reactive free radicals, we can conclude that there are multiple mechanisms of QUIN neurotoxicity. Far from excluding each other, all these factors are somehow closely related and act synergistically to induce axonal damage.

### Axonal Damage

Axonal damage is a major hallmark of MS (Haines et al., 2011). The mechanism of axonal damage in MS is not well understood, but certainly the mitochondrial dysfunction (energy deficit, disrupt of cell homeostasis, etc.) is a key player in it. During the acute inflammatory attack in MS the excitotoxic glutamate and NO produced by activated microglia can damage axonal mitochondria, leading to an imbalance in the energy demands, which can potentiate persistent Na^+^ influx within the axon. To compensate for this redistribution, the Na^+^/Ca^2+^ exchanger instead functions of reverse mode to offset the rising levels of Na^+^, causing increased levels of intracellular Ca^2+^. Dysregulation of Ca^2+^ homeostasis can activate several enzymes, which process leads to axonal damage (Lee et al., 2014). These results suggest that the glutamate excitotoxicity and oxidative/nitrosative stress results in mitochondrial dysfunction, which cause abnormalities of the axonal transport [see review (Su et al., 2009)].

### Cytoskeleton Destabilization

The cytoskeleton plays a key role in maintaining the shape the of neuronal cell and is crucial for its normal functions such as neurite outgrowth, synapse formation, and internal transport of various molecules. Impairment of the cytoskeletal system leads to cell death. There is abundant evidence that QUIN damages the cytoskeletal system, axons, and dendrites (Kerr et al., 1998; Kells et al., 2008). In connection with this, recently published studies have demonstrated that QUIN toxicity can lead to phosphorylation of structural proteins and consequent destabilization of the cytoskeleton (Rahman et al., 2009; Pierozan et al., 2010). During acute intrastriatal injection of QUIN into human primary fetal neurons, NMDA mediated calcium inflow and oxidative stress leading to the phosphorylation of intermediate filaments of striatal nerve cells has been observed (Rahman et al., 2009; Lugo-Huitrón et al., 2013). In rat striatum slices, 100 μM QUIN altered cytoskeleton homeostasis in both astrocytes and neurons. In astrocytes the toxic effects of QUIN manifested in calcium inflow via L-type voltage dependent calcium channels (L-VDCC) and via NMDA receptors, while in neurons they manifested via intracellular calcium channels and metabotropic glutamate receptors. As a result, both cell types show a similar cascade of events: caspase-3 activity is increased, domains in the neurofilament subunits and GFAP are phosphorylated and damage to intermediate filaments develop in both glia cells and nerve cells (Pierozan et al., 2010, 2014, 2015), causing apoptotic cell death (Ishidoh et al., 2010). Rahman et al. (2009) show that mechanisms induced by QUIN cause hyperphosphorylation of the tau protein, also leading to structural changes inside the neuron and they have detected a decrease in the serine/threonine protein phosphatase expression and activity as the culprit of increased tau phosphorylation (Rahman et al., 2009). Their results also suggest, that tau hyperphosphorylation caused by QUIN is also a NMDA receptor-mediated process, as glutamate antagonists had an inhibitory effect on tau hyperphosphorylation. Normally, tau promotes the assembly and maintenance of microtubules, but hyperphosphorylated tau has a decreased affinity to microtubules, and also separates normal tau proteins from microtubules, while forming neurofibrillary tangles inside neurons, leading to structural changes in neurons, and quite possibly being one of the key mechanisms leading to neurodegenerative disorders like Alzheimer’s disease (AD) (Rahman et al., 2009). Abnormal tau phosphorylation and accumulation of tau proteins are associated with loss of axons and nerve cells with parallel proportionate axonal damage and MS progression (Anderson et al., 2008; Petzold et al., 2008). These findings suggest that QUIN exerts a toxic effect on the cytoskeleton mainly through NMDA receptor cascade and is closely related to neurodegeneration. Furthermore, the results of another study suggest, that in addition to QUIN, other KP metabolites may be associated with disturbance of the neuronal cytoskeletal proteins (NfL) (Chatterjee et al., 2019).

## Neurofilaments as a Target of QUIN Toxicity in MS

Kynurenine pathway is a main route of TRP degradation. Pro-inflammatory cytokines can influence IDO-1 activation and thus significantly modify this process. The previous sections provided detailed descriptions of how this process affects the cells of the immune system, and additionally that neurotoxic metabolites produced in the process can inflict direct damage on the cells of the CNS (microglia cells, astrocytes, and oligodendrocytes) and cause glutamate excitotoxicity, leading to mitochondrial dysfunction, free radical formation, destabilization of the cytoskeletal system and ultimately to axonal damage [see review (Lovelace et al., 2016)].

Neuro-axonal damage is a main pathogenic factor in many neurological disorders, therefore a biomarker that is specific for axonal damage could be invaluable in the diagnosis of neurodegenerative conditions, determining the extent of the disease, and monitoring treatment efficacy. NFs could be adequate candidates for this purpose, as they are expressed exclusively in neurons and their level closely mirror the extent of axonal damage and neuronal cell death.

Neurofilaments are intermediate filaments (classified by their diameter, ∼10 nm) specific for neurons, and they establish the neuronal cytoskeleton together with microfilaments and microtubules, thereby providing a structural role in axons and axonal transport (Khalil et al., 2018). NFs are usually composed of the following three subunits: the neurofilament light chain (NfL, 68 kDa), medium chain (NfM, 160 kDa) and heavy chain (NfH, 205 kDa), and they can also include α-internexin (66 kDa) and peripherin (58 kDa) (Gentil et al., 2015; Laser-Azogui et al., 2015; Khalil et al., 2018). NF proteins have a structure that is characteristic for intermediate filaments: they consist of an N-terminal head domain, that is a short, variable region; a central α-helical rod domain, that is relatively conserved; and a C-terminal tail domain of highly variable length (Gentil et al., 2015; Khalil et al., 2018). The head domain contains serine and threonine residues and has multiple phosphorylation and glycosylation sites, while the dominant amino acids in the tail domain are lysine and serine, which also provide multiple phosphorilation sites, and the length of the tail domain is highly characteristic of the neurofilament protein subunit (Khalil et al., 2018). The central rod domain contains hydrophobic heptad repeats, that facilitate the head-to-tail alignment of NF proteins forming coil-to-coil dimers (Gentil et al., 2015; Khalil et al., 2018). This is the first step of the formation of neurofilament heteropolymers: after the formation of dimers, they form tetramers by antiparallel aggregation, then eight tetramers join laterally, creating the cylindrical unit-length filament (ULF) structure (Khalil et al., 2018). The next step is the longitudinal elongation of the NF by the annealing of ULFs, which is followed by radial compaction to form the final NF structure with the diameter of 10 nm (Khalil et al., 2018). The core of the NF consists of NfL subunits, while the NfM and NfH subunits are arranged peripherally, their tails containing multiple phosphorylation sites projecting out radially from the filament structure (Pierozan and Pessoa-Pureur, 2018). NFs are synthesized in the cell body and they are phosphorylated after being transported to the axon.

The exact function of NFs is not yet fully understood, but it is established, that NFs are structural proteins, therefore they have an important role in neuronal organization. NFs provide an intracellular network that protects neurons from mechanical stress, and together with other cytoskeletal elements takes part in creating the intracellular environment and the positioning of organelles, such as the nucleus, axonal mitochondria and the endoplasmic reticulum (ER) (Gentil et al., 2015). NFs are also integral in the radial growth and stability of axons, the maintenance of the axonal diameter and the myelinization, which are the main determinants for efficient, rapid and high–speed nerve conduction. The functions associated with NFs seem to be dependent upon the formation of filamentous structures, however, it is yet undetermined, if they have any functions independent of this process, and neurofilament proteins may have individual functions besides being constituents of NFs (Gentil et al., 2015).

As mentioned earlier, NFs have multiple phosphorylation sites, with the tail domains of NfH and NfM proteins containing many of these sites protruding from the surface of the filament structure. Phosphorylation-dephosphorylation of proteins are an important regulatory mechanism, where negatively charged phosphate groups are added to or removed from Ser, Thr, and Tyr amino acids, thereby changing the function of the protein (Pierozan and Pessoa-Pureur, 2018). The phosphorylation process is executed by protein kinases, while the dephosphorylation is catalyzed by phosphatases. The phosphorylation sites of NfM and NfH subunits are found at repeating Lys-Ser-Pro (KSP) regions at their C-termini (Gentil et al., 2015; Pierozan and Pessoa-Pureur, 2018). The phosphorylation of NFs is a highly specific process catalyzed by mitogen-activated protein kinases (MAPK), such as ERK1/2, JNK, p38MAPK, and proline-directed kinases (e.g., Cdk5), and glycogen synthase kinase 3 (GSK3). The phosphorylation of the NfH tail domain sites most probably plays a role in the regulation of neurofilament mediated axonal transport (Pierozan and Pessoa-Pureur, 2018), and increases the resistance of these subunits against protease activity (Khalil et al., 2018). The N-terminal of the NfL subunit also contains important phosphorylation sites, as the addition of phosphate molecules on these sites regulates the equilibrium of assembly and disassembly of NfM and NfH subunits. The enzymes catalyzing the phosphorylation of NfL head domain sites are c-AMP dependent protein kinase (PKA), protein kinase C (PKC), and Ca^2+^/calmodulin-dependent protein kinase II (PKCaMII) (Pierozan and Pessoa-Pureur, 2018).

Under normal conditions, the phosphorylation-dephosphorylation processes of NFs are in equilibrium, however, an imbalance of these mechanisms can be detected under neurodegenerative conditions. While the NFs with normal phosphorylation are located in the distal parts of the axons, the hyperphosphorylated NFs can form phospho-neurofilament aggregates, which are usually found in the proximal part of axons and the cell body (Pierozan and Pessoa-Pureur, 2018). The accumulation of these aggregates in the cell body can have a cytotoxic effect, and the hyperphosphorylation can affect their interaction with other cytoskeletal components (Pierozan and Pessoa-Pureur, 2018). The misregulation of both kinase and phosphatase activity can lead to the inequilibrium of phosphorylation-dephosphorylation, which can be induced by stress, as well as a number of metabolites that can accumulate in the brain. Studies have shown that one such metabolite is QUIN. QUIN is one of the metabolites produced in the KP, that is the main metabolic pathway of TRP, whose end product is NAD^+^. The enzymes of the KP are mostly located in glial cells in the brain, therefore QUIN is produced predominantly in microglia (Pierozan and Pessoa-Pureur, 2018).

Quinolinic acid is a neuroactive metabolite, that can upset the balance of cytoskeletal homeostasis in neurons by causing abnormal phosphorylation, leading to cell dysfunction and neurodegeneration. As an endogenous NMDA receptor agonist, QUIN activates the NMDA receptors, inducing Ca^2+^ influx into cells, activating phosphorylation enzymes, and consequently causing phosphorylation of cytoskeletal elements, including NFs (Pierozan et al., 2010). Pierozan et al. (2010, 2012) described, that intrastriatal QUIN administration in rat brain induced hyperphosphorylation of NFs in the short term, which effect was mediated by the influx of calcium via NMDA receptor channels under oxidative stress. The hyperphosphorylation of neurofilament was associated with protein kinase PKCaMII, PKA, and PKC activity (Pierozan et al., 2010; Chatterjee et al., 2019), and led to the destabilization of the neurofilament structure. The acutely injected QUIN altered neurofilament phosphorylation in a selective manner, progressing first from the striatum to the cerebral cortex, then to the hippocampus (Pierozan and Pessoa-Pureur, 2018). The activated enzymes catalyzing the phosphorylation differed in the different brain structures: while PKA and PKCaMII were responsible for hyperphosphorylation in the striatum and cortex, MAPKs (such as ERK1/2, JNK, and p38MAPK) were only activated in the hippocampus (Pierozan and Pessoa-Pureur, 2018). This suggests that MAPKs do not take part in the acute toxicity caused by QUIN, they do, however, have a role in the long-lasting effects. According to these findings, the accumulation neurofilament aggregates created by the phosphorylation of NfM and NfH KSP repeats causes disturbances in the axonal transport, that leads to neural dysfunction and behavioral disturbances in the acute phase, followed by motor impairments in the long term, suggesting the role of the hippocampal involvement (Pierozan and Pessoa-Pureur, 2018).

A study made also by Pierozan et al. on acute striatal slices provides further insight into the cellular mechanisms of excitotoxicity caused by QUIN. In neuronal cells, QUIN activated metabotropic glutaminerg receptors (mGluR1 and mGluR5), caused Ca^2+^ influx through NMDA receptors, as well as initiating Ca^2+^ release from the ER. These events caused the downstream activation of the enzymes PKA, PKC, and PKCaMII, which phosphorylate the N-terminal sites of NfL (Pierozan and Pessoa-Pureur, 2018). The activation of the mGluR1 contributes to these actions: it is upstream from phospholipase C (PLC), which produces diacylglycerol (DAG) and inositol-3-phosphate (IP3). DAG contributes to the activation of PKC, while IP3 contributes to the release of Ca^2+^ from intracellular stores, leading to the phosphorylation of KSP repeats at the C-terminals of NfH and NfM subunits. The main enzyme catalyzing the phosphorylation of these sites, however, seems to be Cdk5, which is located downstream of mGluR5 (Pierozan and Pessoa-Pureur, 2018). The misregulation of these signaling pathways caused by QUIN therefore leads to the hyperphosphorylation of neurofilament subunits, which alters the neuronal homeostasis and the structure of the neuronal cytoskeleton, that can be detected by the changed neuron/neurite ratio and neurite outgrowth (Pierozan and Pessoa-Pureur, 2018).

The evidences shown above contribute to the suggestion, that NFs can have a significant role in the development of neurological disorders, as well as being excellent candidates for a biomarker of certain neurological conditions (Khalil et al., 2018). It is known that axonal damage releases NFs into the extracellular space and, consequently, to the CSF and blood, therefore an appropriately sensitive analytical method can be used to detect and quantify neurofilament concentration. It is relatively difficult, because NfL levels in the serum are relatively low, but in the recent years, the fourth generation single molecule array (Simoa) assay technologies presented the possibility to reliably quantify NfL levels in the serum, and detect even minor changes caused by aging or minor injuries (Khalil et al., 2020). There is good correlation between neurofilament levels in the serum and the extent of axonal damage; however, it is important to emphasize that even in healthy controls the blood level of NFs increases by an average of 2.2% per year, and metabolic alterations of neurofilament turnover can also affect the serum level (Khalil et al., 2018). Moreover, there are no standard reference values or intervals of NfL levels established yet, therefore the interpretation of the measured serum NfL levels is precarious at the time being. Elevated neurofilament levels have been detected in various diseases associated with axonal damage, including amyotrophic lateral sclerosis (ALS), AD, PD, stroke, HD, and MS (Khalil et al., 2018). This suggests that NFs are not specific to certain neurological diseases, therefore an elevated neurofilament level might indicate the need for thorough differential diagnosis.

Pathology of MS characterized by neurodegeneration and axonal injury, thereby NfL may be a putative biomarker for determination of neuronal damage. While the standard diagnostic tool for MS is currently MRI, it has its limitations: while it accurately shows lesions in the white matter, lesions in the gray matter are more difficult to detect; moreover, the traditional imaging cannot accurately determine the extent of neuro-axonal degradation, which is the most important factor in determining long-term functional disability (Khalil et al., 2018). In several studies, an elevated neurofilament level in CSF was observed in MS (Malmeström et al., 2003; Rejdak et al., 2008). Recently, an intensive study has been conducted to investigate to presence of NFs protein levels in CSF, as an increase NfL level has been observed in patients not only with RRMS (Lycke et al., 1998), but the NfL levels also seem to correlate well with PPMS (Pawlitzki et al., 2018). Based on immunoassay studies, the CSF levels of NfL correlated well with three aspects of MS: the degree of disability, the disease activity and the time passed from the last relapse in RRMS (Khalil et al., 2018). Recently, based on numerous studies, NfL is considered a promising and reliable prognostic factor for patients with MS, as it well reflects the degree of disease activity, the NfL levels correlate with clinically definitive MS transformation (Kuhle et al., 2015a), they correlate with atrophy of the brain and spinal cord, the relapse rate, or worsening of disability (Kuhle et al., 2016; Petzold et al., 2016; Barro et al., 2018). A long-term follow-up study has found a close correlation between serum NfL levels and MRI lesions and degree of atrophy measured 10 years later (Chitnis et al., 2018), while another found that patients with higher baseline serum NfL levels showed significantly more brain and spinal cord volumes over the 2 and 5 years follow-up (Khalil et al., 2018). Based on a study, patients with ongoing disease-modifying therapy (DMT) had significantly lower serum NfL levels, than untreated patients (Khalil et al., 2018), in line with this, Kuhle et al. (2015b) have determined that fingolimod therapy is associated with significantly lower blood NfL concentration. There is some evidence that KP metabolites can lead to neuro-axonal damage. A recently published study (Chatterjee et al., 2019) confirmed a positive association between KP metabolites, plasma NfL levels and amyloid-β concentration, possibly indicating that a high level of KP metabolites may be associated with NfL damage. Our own recently published study also provides support for this association. We have investigated the correlation and association between biomarkers of neurodegeneration (NfL) and kynurenine metabolites in the CSF of MS patients. Additionally, we have identified a strong positive correlation between NfL and QUIN levels (Rajda et al., 2020). Based on these results it can be concluded that QUIN destabilizes the neuronal cytoskeleton system via the phosphorylation of structural proteins which ultimately leads to neuro-axonal damage, contributing to neurodegeneration in MS. This evidence raises the exciting possibility that NFs could be a new target of neurotoxic QUIN; however, further studies are required to confirm this.

## Conclusion

Several studies have demonstrated that the profile of KP changes in the course of MS progression and that changes in KP metabolites can lead to neuro-axonal damage through multiple mechanisms. Additionally, NFs are good biomarkers of neuro-axonal damage and their levels closely correlate with the extent of damage. These results suggest on one hand that kynurenines are highly relevant to the process of neurodegeneration, and on the other hand that the metabolic profiling of the KP and neurofilament may be potentially useful in finding patients at risk of progression or worse disease outcome, and could be useful in devising individual therapeutic approaches in the future.

## Author Contributions

DP and HP contributed to the writing of the article. CR and LV revised the manuscript. All authors contributed to the article and approved the submitted version.

## Conflict of Interest

The authors declare that the research was conducted in the absence of any commercial or financial relationships that could be construed as a potential conflict of interest.
